# Trends of Visual Impairment and Blindness in the Singapore Chinese Population over a Decade

**DOI:** 10.1038/s41598-018-30004-9

**Published:** 2018-08-15

**Authors:** Yih-Chung Tham, Sing-Hui Lim, Yuan Shi, Miao-Li Chee, Ying Feng Zheng, Jacqueline Chua, Seang-Mei Saw, Paul Foster, Tin Aung, Tien Yin Wong, Ching-Yu Cheng

**Affiliations:** 1 Singapore Eye Research Institute, Singapore National Eye Centre, Singapore, Singapore; 2 Duke-NUS Medical School, Singapore, Singapore; 3 Saw Swee Hock School of Public Health, National University of Singapore and National University Health System, Singapore, Singapore; 4 Institute of Ophthalmology, University College London, London, United Kingdom; 5 Department of Ophthalmology, Yong Loo Lin School of Medicine, National University of Singapore, Singapore, Singapore

## Abstract

We evaluated the prevalence of visual impairment (VI) and blindness among Chinese adults in the Singapore Chinese Eye Study (SCES, 2009–2011), and compared the trends with the Tanjong Pagar Survey, Singapore (TPS), conducted a decade earlier. The SCES comprised of 3,353 Chinese adults aged ≥40 years (response rate, 72.8%). Participants underwent standardized examinations, including measurements of presenting, and best-corrected visual acuity (VA). Bilateral VI (VA < 20/40 to ≥20/200) and blindness (VA < 20/200) were defined based on the United States definition (better-seeing eye). Age-standardized prevalence was calculated using the 2010 Singapore Chinese Population Census. Primary causes and factors associated with VI and blindness were evaluated. In SCES, the age-standardized prevalence of presenting bilateral VI and blindness were 17.7% and 0.6%, respectively; the age-standardised prevalence of best-corrected bilateral VI and blindness were 3.4% and 0.2%, respectively. The previous TPS reported similar rates of best-corrected bilateral VI (3.8%) and blindness (0.3%). In SCES, cataract remains the main cause for both best-corrected bilateral VI (76.0%) and blindness (50.0%). Older age, female, lower income, lower educational level, and smaller housing type were associated with presenting bilateral VI or blindness (all *P* ≤ 0.025). These findings will be useful for the planning of eye care services and resource allocation.

## Introduction

The estimated number of people visually impaired in the world stands at 285 million, with 39 million diagnosed blind. Asia alone accounts for 63.4% and 57.5% of worldwide visual impairment (VI) and blindness, respectively^1^. As Chinese account for 20% of the world population, with a global estimate of more than one billion persons^2^, data on VI and blindness on this ethnic group is important. In this regard, several population-based surveys previously provided considerable information on the trends of VI among Chinese living in both rural and urban parts of China^3–5^. Nevertheless, the prevalence and causes of VI among migrant Chinese who live outside of China are lacking^6^.

In Singapore, Chinese make up the largest ethnic group and currently account for 74.3% of the Singapore population^7^. In the 1990s, the Tanjong Pagar Survey (TPS) was conducted to determine the prevalence of blindness and VI in the Singapore Chinese population^8^. Over the past one decade, socioeconomic conditions and healthcare services in Singapore have improved markedly. Furthermore, the Singapore population is aging rapidly, with a predicted figure of 1 in 5 residents being 65 years old or older by 2030^7^. Hence, the trends of VI and blindness among Singapore Chinese may also change considerably over the years.

The Singapore Chinese Eye Study (SCES) was conducted between 2009–2011 with the key objective to obtain contemporary information on the prevalence of VI and blindness, their causes and key risk factors. The purpose of this current report was to evaluate the prevalence rates and causes of VI and blindness among Chinese adults in the SCES, and to compare the trends with the TPS, conducted a decade earlier. This information will help to improve the understanding on the long-term trends of blinding ocular diseases in Chinese adults residing in Singapore, and will be useful for the future planning of health services and preventive programs for VI and blindness.

## Methodology

### Study Design

The SCES is a population-based, cross-sectional study of 3,353 Chinese adults aged 40 years or older conducted from February 9, 2009, through December 19, 2011. Details of the study design, sampling plan, and methods have been reported previously in detail^9^. The study was conducted in the south-western part of Singapore. Based on age-stratified random 4,605 individuals were identified to be eligible, of which, 3,353 individuals (response rate of 72.8%) were eventually recruited and participated in the study. The study adhered to the Declaration of Helsinki. Ethics approval was obtained from the institutional review board of the Singapore Eye Research Institute, Singapore, and written informed consent was obtained from all participants.

On the other hand, the TPS is a population-based study conducted from 1997 to 1998, on Chinese adults aged 49 to 80 years living in the Tanjong Pagar district (central southern part of Singapore)^8^. Of the 1,717 persons considered eligible to participate, 1,232 participated in the study (response rate of 71.8%), 1,152 had relevant data on visual acuity. Details of the study design and methods of TPS have been described previously^8^. The study adhered to the Declaration of Helsinki. Ethics approval was obtained from Singapore National Eye Centre Ethics Committee, and written informed consent was obtained from all participants^8^.

### Ocular Examination and Visual Acuity (VA) Testing

All study participants underwent a detailed ocular examination including slit lamp examination, intraocular pressure measurement, dilated fundus examination, and fundus photography. The presenting VA (PVA) with habitual correction, and best-corrected VA (BCVA) afer subjective refraction were recorded using an Early Treatment of Diabetic Retinopathy Study logarithm of Minimum Angle of Resolution (logMAR) number chart (Lighthouse International) at a distance of 4 meters^10^. When no numbers could be read at 4 m, the participant was moved to 3, 2, or 1 m, consecutively. When no number could be read at even 1 m, VA was then assessed as counting fingers, hand movements, perception of light, or no perception of light.

### Definitions of VI and Blindness

Both the United States (U.S.) and the modified WHO definitions were used to define VI. According to the U.S. definition, VI was defined as VA < 20/40 but ≥20/200 in the better-seeing eye, and blindness was defined as VA < 20/200 in the better-seeing eye. Based on the WHO definition, VI was defined as VA < 20/60 but ≥20/400 in the better-seeing eye, and blindness was defined as VA < 20/400 in the better-seeing eye. We used a modified WHO definition, which classified persons with VA of counting fingers or worse as blind. All subjects were further categorised into 5 mutually exclusive categories: (1) bilateral blindness; (2) bilateral VI (VI in one eye and VI/blindness in the other eye); (3) unilateral blindness; (4) unilateral VI; and (5) bilateral normal vision. Unilateral VI and unilateral blindness cases were defined based on the worse-seeing eye, with the fellow eye having normal vision.

### Causes of VI and Blindness

Primary causes of VI or blindness were ascertained on the basis of clinical history, examination, disease definition, and clinical judgment. Under-corrected refractive error was defined when BCVA was at least 2 lines (in logMAR chart) better than PVA. Glaucoma was diagnosed according to the International Society of Geographical and Epidemiological Ophthalmology scheme^11^. Age-related macular degeneration (AMD) was graded from retinal photographs using the Wisconsin Age-related Maculopathy grading system^12^. Diabetic retinopathy (DR) was graded from retinal photographs using a modification of the Arlie House classification system for the Early Treatment Diabetic Retinopathy Study^13^. If there were more than one condition in the same eye or across both eyes, the main cause of visual impairment was further determined, based on the severity of the underlying eye diseases.

### Other Measurements

Detailed interviewer-administered questionnaires were also performed to collect relevant medical history (e.g. hypertension, diabetes mellitus, cardiovascular disease, chronic kidney disease), socio-economic status information (e.g. education, income levels, type of housing), and lifestyle-related information (e.g. cigarette use, alcohol consumption) as reported previously^9^.

Systolic and diastolic blood pressures (BP) were measured using an automated sphygmomanometer (DinamapPro100V2; GEHealthCare). Non fasting blood samples were extracted from participants to determine levels of serum glucose and glycosylated haemoglobin (HbA1C). Body mass index was also measured. Patients with hypertension were defined as having systolic BP ≥ 140 mmHg, diastolic BP ≥ 90 mmHg, use of antihypertensive medications, or self-reported physician diagnosed hypertension. Diabetes was defined as having random glucose level of ≥11.1 mmol/L, HbA1c of ≥6.5 mmol/L, use of diabetic medications, or self-reported physician diagnosed diabetes.

### Statistical Analysis

All statistical analyses were performed using R version 2.15.3 (R Development Core Team, 2013, Vienna, Austria). Prevalence estimates of VI and blindness were calculated, and standardized to the Singapore Chinese Population 2010 Census. Bootstrapping was further performed to compare the age-standardized prevalence rates between the SCES and TPS. Multiple logistic regression analysis was used to assess the association between demographic, socioeconomic, and systemic factors with presenting and best-corrected bilateral VI or blindness (U.S definition) in SCES.

### Data Availability

The datasets generated and/or analysed during the current study are not publicly available due to local Institutional Review Board regulation, but are available from the corresponding author on reasonable request.

## Results

Table 1 illustrates the characteristics of study population in SCES. Individuals with presenting bilateral VI or blindness (U.S definition) were older, more likely to have diabetes, hypertension, cardiovascular disease, chronic kidney disease, resided in smaller housing type, and had lower income or education level, compared with individuals without VI (all P < 0.001).

**Table 1:** Characteristics of Participants in the Singapore Chinese Eye Study.

	Persons with presenting normal vision* (N = 2636)	Persons with presenting bilateral visual impairment or blindness* (n = 717)	*P* value
Age, year	57.83 (9.0)	66.49 (10.2)	<0.001
Female gender, n (%)	1,305 (49.5)	385 (53.7)	0.049
Body Mass Index, kg/m^2^	23.8 (3.6)	23.3 (3.8)	0.002
Hypertension, n (%)	1,508 (57.3)	536 (74.8)	<0.001
Diabetes, n (%)	431 (16.4)	161 (22.5)	<0.001
Cardiovascular disease, n (%)	156 (5.9)	78 (10.9)	<0.001
Chronic Kidney Disease, n (%)	128 (5.1)	97 (14.6)	<0.001
Smoking status, n. (%)			
Never smoked	1,960 (74.4)	503 (70.2)	0.016
Current smoker	346 (13.1)	96 (13.4)	
Past smoker	328 (12.5)	118 (16.5)	
Alcohol consumption, n (%)	305 (11.6)	61 (8.5)	0.020
Housing, n (%)			
1-2 room public housing flat	41 (1.6)	33 (4.6)	<0.001
3-4 room public housing flat	1,442 (54.8)	496 (69.3)	
≥5-room public housing flat	1,149 (43.7)	187 (26.1)	
Monthly Income status, n (%)			
<S$2000	1,666 (65.2)	616 (88.5)	<0.001
≥S$2000	889 (34.8)	80 (11.5)	
Education, n (%)			
No formal education	437 (16.6)	318 (44.4)	<0.001
Primary school education or above	2,197 (83.4)	398 (55.6)	

Data presented are means (standard deviations) or number (%), as appropriate. S$ = Singapore Dollars.

*Using the U.S definition, normal vision was defined as ≥20/40, and bilateral visual impairment or worse was defined as VA < 20/40, based on better-seeing eye.

In SCES, based on PVA and U.S definition, age-standardised prevalence of bilateral blindness was 0.6% (95% confidence interval [CI], 0.3–0.9%), 17.7% (95% CI, 16.0–19.6%) for bilateral VI, 2.1% (95% CI, 1.6–2.7%) for unilateral blindness, and 21.1% (95% CI, 19.5–22.8%) for unilateral VI (Table 2). Based on BCVA and U.S definition, the age-standardized prevalence of bilateral blindness was 0.2% (95% CI, 0.1–0.4%), 3.4% (95% CI, 2.8–4.4%) for bilateral VI, 1.6% (95% CI, 1.2–2.1%) for unilateral blindness, and 8.2% (95% CI, 7.3–9.1%) for unilateral VI (Table 2).

**Table 2:** Prevalence of Blindness and Visual Impairment (VI) in the Singapore Chinese Eye Study, Based on the United States Definition^*^.

Vision status	Age group, years	Based on Presenting Visual Acuity			Based on Best-Corrected Visual Acuity		
		All (N = 3,353)	Male (N = 1,662)	Female (N = 1,691)	All (N = 3,353)	Male (N = 1,662)	Female (N = 1,691)
		n, (%)	n, (%)	n, (%)	n, (%)	n, (%)	n, (%)
Bilateral blindness	40-49	1 (0.1)	0 (0.0)	1 (0.3)	0 (0.0)	0 (0.0)	0 (0.0)
	50–59	5 (0.5)	3 (0.6)	2 (0.3)	1 (0.1)	1 (0.2)	0 (0.0)
	60–69	4 (0.4)	1 (0.2)	3 (0.7)	2 (0.2)	1 (0.2)	1 (0.2)
	70+	12 (1.9)	4 (1.2)	8 (2.7)	5 (0.8)	2 (0.6)	3 (1.0)
	Total	22 (0.7)	8 (0.5)	14 (0.8)	8 (0.2)	4 (0.2)	4 (0.2)
	**Age-standardised prevalence, % (95% CI)** ^**†**^	**0.6 (0.3–0.9)**	**0.4 (0.2–0.9)**	**0.8 (0.4–1.4)**	**0.2 (0.1–0.4)**	**0.2 (0.1–0.6)**	**0.2 (0.1–0.6)**
Bilateral VI^	40–49	62 (8.0)	26 (7.5)	36 (10.0)	3 (0.4)	1 (0.3)	2 (0.6)
	50–59	125 (11.2)	50 (9.9)	75 (12.3)	6 (0.6)	1 (0.2)	5 (0.8)
	60–69	210 (23.4)	95 (20.1)	115 (27.0)	29 (3.3)	12 (2.6)	17 (4.0)
	70+	298 (47.1)	153 (45.5)	145 (49.0)	112 (17.7)	57 (17.0)	55 (18.6)
	Total	695 (20.7)	324 (19.5)	371 (21.9)	150 (4.5)	71 (4.3)	79 (4.7)
	**Age-standardised prevalence, % (95% CI)** ^**†**^	**17.7 (16.0–19.6)**	**15.3 (13.1–18.0)**	**20.1 (17.6–23.1)**	**3.4 (2.8–4.4)**	**2.7 (1.9–4.0)**	**4.2 (3.0–5.8)**
Unilateral blindness	40–49	12 (1.7)	6 (1.7)	6 (1.7)	5 (0.7)	3 (0.9)	2 (0.6)
	50–59	17 (1.5)	9 (1.8)	8 (1.3)	11 (1.0)	4 (0.8)	7 (1.2)
	60–69	25 (2.8)	17 (3.6)	8 (1.9)	18 (2.0)	12 (2.5)	6 (1.4)
	70+	21 (3.3)	13 (3.9)	8 (2.7)	30 (4.8)	17 (5.1)	13 (4.4)
	Total	75 (2.2)	45 (2.7)	30 (1.8)	64 (1.9)	36 (2.2)	28 (1.7)
	**Age-standardised prevalence, % (95% CI)** ^**†**^	**2.1 (1.6–2.7)**	**2.4 (1.7–3.3)**	**1.8 (1.2–2.6)**	**1.6 (1.2–2.1)**	**1.7 (1.1–2.4)**	**1.5 (1.0–2.3)**
Unilateral VI	40–49	113 (15.9)	58 (16.6)	55 (15.2)	16 (2.3)	9 (2.6)	7 (1.9)
	50–59	237 (21.3)	104 (20.7)	133 (21.9)	39 (3.5)	21 (4.2)	18 (3.0)
	60–69	247 (27.4)	143 (30.2)	104 (24.4)	113 (12.6)	61 (12.9)	52 (12.2)
	70+	157 (24.8)	91 (27.1)	66 (22.3)	174 (27.5)	86 (25.6)	88 (29.7)
	Total	754 (22.5)	396 (23.8)	358 (21.2)	342 (10.2)	177 (10.6)	165 (9.8)
	**Age-standardised prevalence, % (95% CI)** ^**†**^	**21.1 (19.5–22.8)**	**21.8 (19.6–24.3)**	**20.2 (18.0–22.5)**	**8.2 (7.3–9.1)**	**7.8 (6.7–9.2)**	**8.6 (7.3–10.1)**

^*^Based on the U.S definition, VI was defined as VA < 20/40 to ≥20/200. Blindness was defined as VA < 20/200.

^#^Bilateral blindness and bilateral VI were defined based on better-seeing eye, while unilateral blindness and unilateral VI were defined based on worse-seeing eye.

^^^Bilateral VI includes participants with VI in one eye and VI/ blindness in the other eye.

^†^Standardised to Singapore Chinese population 2010 census.

Supplementary Table 1 shows the prevalence of blindness and VI based on WHO definition in SCES. The age-standardised prevalence of presenting bilateral blindness was 0.2% (95% CI, 0.1–0.4%), 7.8% (95% CI, 6.7–9.2%) for bilateral VI, 1.7% (95% CI, 1.3–2.2%) for unilateral blindness, and 13.9% (95% CI, 12.7–15.2%) for unilateral VI. On the other hand, the age-standardised prevalence of best-corrected bilateral blindness was 0.1% (95% CI, 0.0–0.3%), 1.3% (95% CI, 0.9–1.9%) for bilateral VI, 1.5% (95% CI, 1.2–2.0%) for unilateral blindness, and 3.7% (95% CI, 3.1–4.4%) for unilateral VI.

Table 3 compares the prevalence of blindness and VI in TPS with SCES. In TPS, based on BCVA and U.S definition, the age-standardised prevalence of bilateral blindness was 0.3% (95% CI, 0.1–0.9%), and bilateral VI was 3.8% (95% CI, 2.9–5.0%). Based on BCVA and WHO definition, age-standardised prevalence of bilateral blindness was 0.2% (95% CI, 0.1–0.8%), and bilateral VI was 1.6% (95% CI, 1.0–2.5%). Overall, when compared with TPS, the prevalence of best-corrected blindness and VI in SCES were slightly lower but the difference was not statistically significant.

**Table 3:** Comparison between the Tanjong Pagar Survey (TPS) and the Singapore Chinese Eye Study (SCES) on the prevalence of best-corrected blindness and visual impairment (VI).

Vision Status^#^	**Age group, years**	**United States Definition**†	**World Health Organization Definition**‡		
		**TPS (N** = **1,152)**	**SCES (N = 3,353)**	**TPS (n = 1,152)**	**SCES (n = 3,353)**
		n, (%)	n, (%)	n, (%)	n, (%)
Bilateral blindness	40–49	0 (0.0)	0 (0.0)	0 (0.0)	0 (0.0)
	50–59	0 (0.0)	1 (0.1)	0 (0.0)	0 (0.0)
	60–69	1 (0.3)	2 (0.2)	0 (0.0)	1 (0.1)
	70 +	5 (2.0)	5 (0.8)	4 (1.6)	3 (0.5)
	Total	6 (0.5)	8 (0.2)	4 (0.3)	4 (0.1)
	Age-standardised prevalence, % (95% CI)*	**0.3 (0.1–0.9)**	**0.2 (0.1–0.4)**	**0.2 (0.1–0.8)**	**0.1 (0.0–0.3)**
Bilateral VI^	40–49, n (%)	1 (0.4)	3 (0.4)	1 (0.4)	1 (0.1)
	50–59, n (%)	3 (1.1)	6 (0.5)	2 (0.7)	4 (0.4)
	60–69, n (%)	20 (6.5)	29 (3.2)	8 (2.6)	9 (1.0)
	70+, n (%)	39 (15.2)	112 (17.7)	14 (5.5)	42 (6.7)
	Total, n (%)	63 (5.5)	150 (4.5)	25 (2.2%)	56 (1.7)
	Age-standardised prevalence, % (95% CI)*	**3.8 (2.9–5.0)**	**3.4 (2.9–4.0)**	**1.6 (1.0–2.5)**	**1.3 (0.9–1.9)**

^†^Based on the U.S definition, VI was defined as VA < 20/40 to ≥20/200. Blindness was defined as VA < 20/200.

^‡^Based on the WHO definition, VI was defined as VA < 20/60 to ≥20/400. Blindness was defined as VA < 20/400.

^**#**^Bilateral blindness and bilateral VI were defined based on better-seeing eye.

^^^Bilateral VI includes participants with VI in one eye and VI/ blindness in the other eye.

*Standardised to Singapore Chinese adult population 2010 census.

Table 4 shows the causes of VI and blindness (U.S definition) in SCES. Based on PVA, the leading causes of bilateral VI were under-corrected refractive error (58.6%), cataract (33.2%), AMD (1.4%) and DR (1.4%). On the other hand, cataract was the leading cause of presenting bilateral blindness (40.9%), followed by under-corrected refractive error (22.7%) and AMD (18.2%). After refractive correction (i.e. based on BCVA), the leading causes of bilateral VI were cataract (76.0%), followed by DR (5.3%), myopic maculopathy (4.7%), and posterior capsular opacification (4.7%). Cataract remained to be the primary cause of best-corrected bilateral blindness (50.0%).

**Table 4:** Causes of Visual Impairment (VI) and Blindness in the Singapore Chinese Eye Study, Based on the United States Definition.

Causes	Based on Presenting Visual Acuity		Based on Best-Corrected Visual Acuity	
	Bilateral VI (N = 695)^	Bilateral Blindness, (N = 22)	Bilateral VI (N = 150)^	Bilateral Blindness (N = 8)
	n, (%)	n, (%)	n, (%)	n, (%)
Under-corrected refractive error	407 (58.6)	5 (22.7)	0 (0.0)	0 (0.0)
Cataract	231 (33.2)	9 (40.9)	114 (76.0)	4 (50.0)
Age-related macular degeneration	10 (1.4)	4 (18.2)	4 (2.7)	2 (25.0)
Diabetic retinopathy	10 (1.4)	2 (9.1)	8 (5.	1 (12.5)
Posterior capsular opacity	9 (1.3)	0 (0.0)	7 (4.7)	0 (0.0)
Myopic maculopathy	5 (0.7)	1 (4.5)	7 (4.7)	0 (0.0)
Glaucoma	5 (0.7)	0 (0.0)	0 (0.0)	0 (0.0)
Epiretinal membrane	5 (0.7)	0 (0.0)	4 (2.7)	0 (0.0)
Corneal opacity	2 (0.3)	1 (4.5)	0 (0.0)	1 (12.5)
Pterygium	1 (0.1)	0 (0.0)	1 (0.7)	0 (0.0)
Ambylopia	1 (0.1)	0 (0.0)	2 (1.3)	0 (0.0)
Retinal vein occlusion	0 (0.0)	0 (0.0)	1 (0.7)	0 (0.0)
Others	9 (1.3)	0 (0.0)	2 (1.3)	0 (0.0)

^^^Bilateral VI includes participants with VI in one eye and VI/blindness in the other eye.

Table 5 shows demographic, socioeconomic, and systemic factors associated with presenting and best-corrected bilateral VI or blindness (U.S definition) in SCES. Multiple logistic regression model demonstrated that older age (per decade, odds ratio [OR] = 2.00; 95% CI, 1.76–2.27), female (OR = 1.31; 95% CI, 1.04–1.67), smaller housing type (those residing in 1–2 room public flats, OR = 2.58; 95% CI, 1.48–4.49), lower income (OR = 1.64; 95% CI, 1.23–2.19), and no formal education (OR = 1.66; 95% CI, 1.32–2.66) were significantly associated with presenting bilateral VI or blindness. For best-corrected bilateral VI or blindness, older age (per decade, OR = 3.66; 95% CI, 2.77–4.83), no formal education (OR = 1.72; 95% CI, 1.12–2.66), and diabetes (OR = 1.59; 95% CI, 1.04–2.44) were significant associated factors.

**Table 5:** Factors Associated with Presenting, and Best-corrected Bilateral Visual Impairment (VI) or Blindness (United States Definition).

	Presenting Bilateral VI or Blindness as outcome (N = 717)		Best-corrected Bilateral VI or Blindness as outcome (N = 158)	
	Odds Ratio (95% CI)	P value	Odds Ratio (95% CI)	P value
Age (per decade)	2.00 (1.76–2.27)	<0.001	3.66 (2.77–4.83)	<0.001
Female	1.31 (1.04–1.67)	0.025	1.34 (0.82–2.19)	0.237
**Medical Conditions**				
Hypertension	0.92 (0.73–1.16)	0.490	0.98 (0.56–1.73)	0.956
Diabetes Mellitus	0.99 (0.78–1.26)	0.941	1.59 (1.04–2.44)	0.033
Cardiovascular Disease	1.10 (0.77–1.57)	0.598	1.27 (0.72–2.23)	0.407
Chronic Kidney Disease	1.26 (0.90–1.75)	0.174	1.19 (0.72–1.97)	0.497
**BMI category**				
Normal BMI	Reference		Reference	
Underweight	1.19 (0.80–1.76)	0.395	1.08 (0.53–2.22)	0.825
Overweight	0.89 (0.71–1.12)	0.336	0.87 (0.67–1.12)	0.078
Obese	0.83 (0.53–1.30)	0.418	0.92 (0.38–2.19)	0.872
**Smoking category**				
Never-smoked	Reference		Reference	
Current	1.33 (0.96–1.92)	0.082	1.47 (0.77–2.78)	0.243
Past	1.19 (0.88–1.63)	0.264	1.25 (0.71–2.23)	0.439
Alcohol Consumption	0.93 (0.66–1.32)	0.696	1.35 (0.67–2.72)	0.408
**Housing type**				
≥5 room public housing flat	Reference		Reference	
3–4 room public housing flat	1.50 (1.21–1.87)	<0.001	1.23 (0.78–1.96)	0.373
1–2 room public housing flat	2.58 (1.48–4.49)	0.001	2.00 (0.83–4.80)	0.123
**Monthly Income category**				
≥S$2000	Reference		Reference	
<S$2000	1.64 (1.23–2.19)	0.001	2.73 (0.95–7.86)	0.063
**Education category**				
Formal education	Reference		Reference	
No formal education	1.66 (1.32–2.08)	<0.001	1.72 (1.12–2.66)	0.014

CI = Confidence Interval; BMI = Body mass index; S$ = Singapore Dollars.

## Discussion

We evaluated the prevalence and causes of VI and blindness among Chinese adults in the SCES, and compared the trends with a previous Singapore Chinese population study (TPS) conducted a decade ago. In SCES, 17.7% and 0.6% had presenting bilateral VI and blindness (U.S. definition), respectively; while 3.4% and 0.2% had best-corrected bilateral VI and blindness (U.S definition), respectively. These rates in SCES were slightly lower compared to those in the previous TPS. The principal cause of best-corrected bilateral VI and blindness was cataract. This is a unique study which provided rare formation on the long-term change in prevalence of VI and blindness in an urban Chinese population, over a decade. Collectively, these findings will be useful for the future designing of eye health services, and will help to better guide planning in health resource allocation.

The slightly lower prevalence of VI and blindness in the current SCES compared to TPS, may be attributed to increased awareness of eye health and eye diseases, and enhanced accessibility to eye care services in Singapore, over the past decade. This explanation is further substantiated with the reported increase in eye care utilisation in Singapore over the years, with an annual growth rate of 8.6% per year since year 2002^14^.

In addition, when comparing with other population-based studies among Chinese adults in China, Taiwan and Mongolia, the prevalence rates of VI and blindness in SCES are also relatively lower^4,5,15–18^. This comparison is especially marked when comparing with previous studies of rural areas such as the Yunnan Minority Eye Study and Kailu Study^16–18^. Overall, these differences are expected given the differences in accessibility and utilisation of eye care services between Singapore and the above-mentioned areas. Nevertheless, it should also be noted that accurate comparisons across studies is limited by the inherent variations in population characteristics, and time period between studies. Thus, these observed differences should also be interpreted with caution.

With regards to causes of visual loss, under-corrected refractive error was the main cause of presenting bilateral VI in SCES. This is consistent with previous Chinese population studies^3,4^. This finding indicates that more public education and awareness enhancement are required in this aspect, in a bid to further reduce the rate of avoidable VI due to uncorrected refractive error. On the other hand, cataract was the most common cause for best-corrected bilateral VI in SCES. In TPS and other Asian populations, it was similarly reported that cataract was the most common cause of best-corrected bilateral VI^8,19^. These collectively indicate that cataract still remains a major burden for best-corrected VI after a decade, further emphasizing the importance of continually improving accessibility to cataract surgery and optimal cataract surgery outcomes, which will in turn help to further reduce cataract-related VI and blindness. In addition, cataract was also the leading cause for best-corrected bilateral blindness in SCES. In contrast, glaucoma was the most common cause for best-corrected bilateral blindness in TPS, and cataract was the second leading cause instead^8^. This difference may be explained by the higher proportion of advanced glaucoma cases observed in TPS back in late 1990s^20^. Approximately 40% of glaucoma cases identified in TPS were of late stage with blindness in at least one eye. Interestingly, a decade later in SCES, no glaucoma case was reported as the primary cause for bilateral blindness (Table 4), This change may be due to enhanced glaucoma awareness, improved early detection and treatment over the years.

Furthermore, we observed that older age and lower educational level were also associated with both presenting, and best-corrected bilateral VI or worse. Previous studies similarly showed that older age was associated with VI, with some reported marked increase in the rate of VI for those aged 70 years and above^4,5,21,22^. Likewise, Beijing Eye Study also reported that lower educational level was associated with VI^5^. One possible explanation might be that individuals with lower educational level may have limited awareness and understanding of their own medical conditions, are less likely to go for routine eye health screening, and more likely of having poorer compliance when referred to tertiary eye care for further treatment^23^. Lastly, lower socioeconomic status such as lower income and smaller housing residence type were found to be associated with presenting bilateral VI or worse. Nevertheless, it should be noted that this association may be bidirectional; while persons with lower economic status may be more likely to suffer from poor vision, it is also likely that VI hinders individual’s earning and career potential.

The strengths of this study include a large sample of urban Chinese population, and the use of standardized examination protocol which was similar to that of TPS. This allowed for relatively direct comparison between the 2 studies, thus providing unique information on the long-term change in prevalence of VI and blindness in Singapore Chinese population. Nevertheless, our study has a few limitations. First, as automated perimetry was only performed on a subset of study participants identified to be glaucoma suspects, loss of visual fields was not included as part of the definition for blindness. Therefore, the prevalence of blindness due to glaucoma may have been underestimated. Second, as cataract accounted for 76.0% of VI after refractive correction, it was possible that concurrent retinal diseases might had been masked by significant cataract, especially among elderly individuals.

In conclusion, the SCES showed relatively low prevalence of VI and blindness among Chinese adults living in Singapore. These estimates are slightly lower compared with previous findings from the TPS conducted a decade earlier. Cataract remains the leading cause of best-corrected bilateral VI and blindness. These findings will further help to improve the understanding on the long-term trends of blinding ocular diseases in Chinese adults residing in Singapore, which are useful for the future planning of health services, resource allocation, and preventive programs for VI and blindness.

## Electronic supplementary material

Supplementary Table 1
