# Supplementary material for: Trends of Visual Impairment and Blindness in the Singapore Chinese Population over a Decade
Source: Sci Rep. 2018 Aug 15;8:12224. doi: 10.1038/s41598-018-30004-9 (PMC6093864; doi:10.1038/s41598-018-30004-9)
Supplement: Supplementary file 1 — Supplementary Table 1 [file 41598_2018_30004_MOESM1_ESM.pdf]

Trends of Visual Impairment and Blindness in the Singapore Chinese Population over a Decade

Yih-Chung Tham, PhD,[1*] Sing-Hui Lim, MD,[1*] Yuan Shi, PhD,[1] Miao-Li Chee, BSc,[1] Ying Feng Zheng, MD, PhD,[1] Jacqueline Chua, PhD,[1,2] Seang-Mei Saw, MBBS, PhD,[1,2,3] Paul Foster, FRCOph, PhD,[4] Tin Aung, PhD, FRCS(Ed),[1,2,5] Tien Yin Wong, PhD, FRCS(Ed), [1,2,5] Ching-Yu Cheng, MD, PhD[1,2,5]

1. Singapore Eye Research Institute, Singapore National Eye Centre, Singapore
2. Duke-NUS Medical School, Singapore
3. Saw Swee Hock School of Public Health, National University of Singapore and National University Health System
4. Institute of Ophthalmology, University College London, United Kingdom
5. Department of Ophthalmology, Yong Loo Lin School of Medicine, National University of Singapore, Singapore

*Contributed equally as first authors.

Dr Ying Feng Zheng is currently with State Key Laboratory of Ophthalmology, Zhongshan Ophthalmic Center, Sun Yat-sen University, Guangzhou, China.

Supplementary Table 1: Prevalence of Blindness and Visual Impairment (VI) Based on the World Health Organization Definition.*

		Based on Presenting Visual Acuity			Based on Best-Corrected Visual Acuity		
	**Age group, years**	**All (N=3,353)**	**Male (N=1,662)**	**Female (N=1,691)**	**All (N=3,353)**	**Male (N=1,662)**	**Female (N=1,691)**
**Vision status#**		n, (%)	n, (%)	n, (%)	n, (%)	n, (%)	n, (%)
Bilateral blindness	40-49	0 (0.0)	0 (0.0)	0 (0.0)	0 (0.0)	0 (0.0)	0 (0.0)
	50-59	0 (0.0)	0 (0.0)	0 (0.0)	0 (0.0)	0 (0.0)	0 (0.0)
	60-69	2 (0.2)	1 (0.2)	1 (0.2)	1 (0.1)	1 (0.2)	0 (0.0)
	70+	5 (0.8)	1 (0.3)	4 (1.4)	3 (0.5)	1 (0.3)	2 (0.7)
	Total	7 (0.2)	2 (0.1)	5 (0.3)	4 (0.1)	2 (0.1)	2 (0.1)
	**Age standardised prevalence, % (95% CI)†**	**0.2 (0.1–0.4)**	**0.1 (0.0–0.5)**	**0.3 (0.1–0.7)**	**0.1 (0.0–0.3)**	**0.1 (0.0–0.5)**	**0.1 (0.0–0.5)**
Bilateral visual impairment^	40-49	27 (3.8)	13 (3.8)	14 (3.9)	1 (0.1)	0 (0.0)	1 (0.3)
	50-59	43 (3.8)	19 (3.8)	24 (3.9)	4 (0.4)	2 (0.4)	2 (0.3)
	60-69	87 (9.7)	42 (8.9)	45 (10.6)	9 (1.0)	4 (0.8)	5 (1.2)
	70+	155 (24.5)	80 (23.9)	75 (25.3)	42 (6.7)	19 (5.7)	23 (7.8)
	Total	312 (9.3)	154 (9.3)	158 (9.3)	56 (1.7)	25 (1.5)	31 (1.8)
	**Age standardised prevalence, % (95% CI)†**	**7.8 (6.7–9.2)**	**7.1 (5.6–9.1)**	**8.5 (6.8–10.8)**	**1.3 (0.9–1.9)**	**1.0 (0.6–1.9)**	**1.7 (1.0–2.9)**
Unilateral blindness	40-49	6 (0.9)	4 (1.2)	2 (0.6)	4 (0.6)	2 (0.6)	2 (0.56)
	50-59	11 (1.0)	2 (0.4)	9 (1.48)	9 (0.81)	1 (0.2)	8 (1.3)
	60-69	25 (2.8)	18 (3.8)	7 (1.64)	18 (2.0)	14 (2.95)	4 (0.9)
	70+	23 (3.6)	16 (4.76)	7 (2.36)	31 (4.91)	21 (6.25)	10 (3.4)
	Total	65 (1.9)	40 (2.4)	25 (1.5)	62 (1.8)	38 (2.3)	24 (1.4)
	**Age standardised prevalence, % (95% CI)†**	**1.7 (1.3–2.2)**	**1.8 (1.3–2.6)**	**1.3 (0.9–2.1)**	**1.5 (1.2–2.0)**	**1.6 (1.1–2.3)**	**1.3 (0.8–2.0)**

Unilateral visual impairment	40-49	60 (8.5)	24 (6.88)	36 (9.97)	9 (1.27)	6 (1.72)	3 (0.83)
	50-59	138 (12.4)	55 (10.93)	83 (13.65)	14 (1.26)	8 (1.59)	6 (0.99)
	60-69	153 (17.0)	86 (18.14)	67 (15.73)	47 (5.22)	28 (5.91)	19 (4.46)
	70+	168 (26.6)	86 (25.6)	82 (27.7)	85 (13.45)	47 (13.99)	38 (12.84)
	Total	519 (15.5)	251 (15.1)	268 (15.8)	155 (4.6)	89 (5.4)	66 (3.9)
	**Age standardised prevalence, % (95% CI)[†]**	**13.9 (12.7–15.2)**	**12.6 (11.0–14.5)**	**15.0(13.2–17.1)**	**3.7 (3.1–4.4)**	**4.0 (3.1–5.0)**	**3.5 (2.7–4.5)**

*Based on the WHO definition, VI was defined as VA <20/60 to ≥20/400. Blindness was defined as VA <20/400.

[#]Bilateral blindness and bilateral VI were defined based on better-seeing eye, while unilateral blindness and unilateral VI were defined based on worse-seeing eye.

^ Bilateral VI includes participants with VI in one eye and VI/ blindness in the other eye.

[†]Standardised to Singapore Chinese population 2010 census.
